# The Role of the Microbiome and of Radiotherapy-Derived Metabolites in Breast Cancer

**DOI:** 10.3390/cancers16213671

**Published:** 2024-10-30

**Authors:** Lourdes Herrera-Quintana, Héctor Vázquez-Lorente, Rafael Cardoso Maciel Costa Silva, Jorge Olivares-Arancibia, Tomás Reyes-Amigo, Bruno Ricardo Barreto Pires, Julio Plaza-Diaz

**Affiliations:** 1 Department of Physiology, Schools of Pharmacy and Medicine, University of Granada, 18071 Granada, Spain; lourdesherrera@ugr.es (L.H.-Q.); hectorvazquez@ugr.es (H.V.-L.); 2 Biomedical Research Center, Health Sciences Technology Park, University of Granada, 18016 Granada, Spain; 3 Medical Sciences Faculty, Universidade do Estado do Rio de Janeiro, Campus Cabo Frio, Rio de Janeiro 28905-320, RJ, Brazil; rcmcs@biof.ufrj.br; 4 AFySE Group, Research in Physical Activity and School Health, School of Physical Education, Faculty of Education, Universidad de Las Américas, Santiago 7500975, Chile; jolivares@udla.cl; 5 Physical Activity Sciences Observatory (OCAF), Department of Physical Activity Sciences, Universidad de Playa Ancha, Valparaíso 2360072, Chile; tomas.reyes@upla.cl; 6 Biometry and Biophysics Department, Institute of Biology Roberto Alcantara Gomes (IBRAG), Universidade do Estado do Rio de Janeiro, Rio de Janeiro 20551-030, RJ, Brazil; bruno.pires@uerj.br; 7 Instituto de Investigación Biosanitaria IBS.GRANADA, Complejo Hospitalario Universitario de Granada, 18014 Granada, Spain; 8 School of Health Sciences, Universidad Internacional de La Rioja, Avenida de la Paz, 137, 26006 Logroño, Spain

**Keywords:** breast cancer, radiotherapy-derived metabolites, microbiome, genomics, dietary factors

## Abstract

The gut microbiome has emerged as a crucial player in modulating cancer therapies, including radiotherapy. In the case of breast cancer, the interplay between the microbiome and radiotherapy-derived metabolites may enhance therapeutic outcomes and minimize adverse effects. In this review, we explore the bidirectional relationship between the gut microbiome and breast cancer. We explain how gut microbiome composition influences cancer progression and treatment response, and how breast cancer and its treatments influence microbiome composition. A dual role for radiotherapy-derived metabolites is explored in this article, highlighting both their therapeutic benefits and potential hazards. By integrating genomics, metabolomics, and bioinformatics tools, we present a comprehensive overview of these interactions. The study provides real-world insight through case studies and clinical trials, while therapeutic innovations such as probiotics, and dietary interventions are examined for their potential to modulate the microbiome and enhance treatment effectiveness. Moreover, ethical considerations and patient perspectives are discussed, ensuring a comprehensive understanding of the subject. Towards revolutionizing treatment strategies and improving patient outcomes, the review concludes with future research directions. It also envisions integrating microbiome and metabolite research into personalized breast cancer therapy.

## Simple Summary

Breast cancer is the most common cancer type and the leading cause of mortality for women worldwide. The microbiome influences various cancer therapies, including radiotherapy. Thus, radiotherapy-derived metabolites and microbiome composition may affect cancer progression and treatment response, and vice versa. This review explores this bidirectional relationship as well as providing current evidence and future perspectives in this field.

## 1. Breast Cancer and the Microbiome: A Bidirectional Relationship

### 1.1. Cancer and the Microbiome

In spite of the fact that the terms “microbiota” and “microbiome” are often used interchangeably, there are some differences between them. The term “microbiota” refers to the living microorganisms, including bacteria, fungi, and viruses, that inhabit a defined environment, such as the oral cavity and gut [1]. The microbiome describes the collection of genomes of all microorganisms found in the environment, which comprises not only the community of microorganisms, but also the structure, metabolites, and environmental conditions of the microorganisms in the environment. Hence, the term microbiome encompasses a broader spectrum than microbiota [2]. This review will use the term “microbiome.” The microbiome affects most physiological functions through direct and indirect processes [3] and the gut microbiome is known to impact beyond gut function, extending its effects to distant organs through mechanisms that involve activating or inhibiting metabolic pathways and producing specific metabolites. These mechanisms include the modulation of immune system responses, metabolite production, and the gut–brain axis [4]. Likewise, most tissues contain a complex ecosystem of microbes that changes and adapts to environmental conditions and influences biological functions [5]. Thus, the diversity and composition of the microbiome, which differs considerably among individuals and populations, significantly influences the development and progression of different diseases, including cancer [4]. Cancer patients have been shown to have altered microbiome composition. There is also evidence that pathogenic microbes harboring specific virulence factors contribute to the development of a variety of cancers, including colorectal, gastric, and pancreatic cancers [6].

#### 1.1.1. Cancer Development and Progression

During cancer development, tumor cells are characterized by the acquisition of six biological capabilities: evading growth suppressors, sustaining proliferative signaling, enabling replicative immortality, resisting cell death, inducing angiogenesis, and activating invasion and metastasis [7].

Cancer is caused by the mutation of cancer susceptibility genes (e.g., oncogenes or tumor suppressor genes), with different factors contributing to this process. These mutations lead to the appearance of malignant cells, characterized by their proliferative capacity, as they can escape from control mechanisms [8]. However, it is important to note that cancer does not occur exclusively in cancer cells. A tumor microenvironment (TME) plays an essential role in the survival and progression of tumors as cancer cells are capable of functionally modifying and reprogramming the surrounding stroma [9]. Significant interactions occur between the extracellular matrix (ECM) and tumor cells during cancer development [10]. The ECM is formed by non-cellular components of the tissue (e.g., proteoglycans and fibrous proteins) and constantly remodels. The ECM’s dynamic biophysical and biochemical characteristics provide essential cell structural support [10].

Cancer development is also affected by inflammation [11,12,13,14,15,16]. A deregulated or maintained inflammatory process can influence the malignant transformation of cells, with an extensive range of inflammatory mediators (e.g., cytokines, prostaglandins, enzymes or matrix metalloproteinases) mediating the creation of a favorable TME [17]. Inflammation leads to vascular hyperpermeability; hence, chronic inflammation may promote pathological angiogenesis during the cancer process. The induction of angiogenesis by tumor cells aims to supply the increased need for nutrients due to the elevated metabolic rate [18]. In addition, inflammatory responses lead to reactive oxygen species (ROS) liberation and chromosome instability, further contributing to tumorigenesis. On the other side, proteases and ROS, at certain levels, can also promote tumor destruction. The major cause of cancer mortality is metastasis. This is defined as the cancer cells’ capacity to detach from the primary tumor, travel through blood vessels, and settle and grow at a distal site. In this process, parameters and events related to TME have particular relevance [19]. The close relationship between inflammation and tumors makes targeting inflammation an important component of anti-cancer treatment [20]. Despite the fact that inhibition of inflammation targeting innate and adaptive immunity has provided remarkable accomplishments in cancer therapy, several obstacles and challenges remain [20]. Inflammation-targeting cancer therapy emphasizes several mechanisms by which inflammation interacts with cancer. The essence of this approach is to promote cancer-inhibiting inflammation and inhibit cancer-promoting inflammation, with the most significant challenge being to maintain the balance of inflammatory responses [20].

#### 1.1.2. Dysbiosis and Cancer

It is well known that gut microbiome composition greatly varies between individuals, being influenced by genetic factors, lifestyle and eating habits, the presence of diseases and the treatment with antibiotics, among other factors [21]. The term dysbiosis refers to changes in the quantitative and qualitative composition of the microbiome, leading to altered host–microbial interactions and altered disease states. As a consequence, antigens and metabolites may be released, resulting in immune dysregulation and chronic inflammation [22,23]. These disturbances appear to have a key role in different tumorigenesis processes [24]. There is evidence that colorectal cancer has been linked to dysbiosis involving specific bacterial species, such as *Fusobacterium nucleatum*, *Escherichia coli*, and *Bacteroides fragilis*, as well as disruption of virome commensals [25]. Another study has indicated that esophageal squamous dysplasia and esophageal squamous cell carcinoma patients have enriched *Clostridium* species in their microbiome. This suggests that gastric dysbiosis plays a significant role in the progression from esophageal squamous dysplasia to squamous cell carcinoma [26].

At present, substantial evidence links gut microbiome dysbiosis with the development of certain types of cancer, especially relevant to colorectal cancer. However, investigating and demonstrating the causal role of these microbes in cancer processes is still needed [25].

Likewise, it has been hypothesized that gut microbiome metabolites may affect the TME of distant cancers [21]. Furthermore, the gut microbiome seems to influence cancer treatments’ response, for instance, decreasing immunotherapy response. In this line, it has been observed that low levels of *Akkermansia muciniphila* are common in nonresponding patients with cancer, thereby making oral supplementation with this bacterium a therapeutic strategy, one which has shown promising results in mice models [27]. *Akkermansia muciniphila* affects immune cells’ composition and enhances immune regulation by regulating pleiotropic cytokines, including interferon (IFN)-γ, tumor necrosis factor (TNF)-α, Th17, interleukin (IL)-10, IL-33, and IL-4, with multiple immunomodulatory effects [28,29,30,31]. Thus, understanding the underlying mechanisms by which the microbiome affects cancer development and treatment response is of significant importance.

### 1.2. Breast Cancer and the Microbiome

#### 1.2.1. Breast Cancer Overview

Breast cancer (BC) is a major health concern among women due to its high morbidity and mortality rate [32]. According to the World Health Organization (WHO), in 2022, BC caused 670,000 deaths, was the most common cancer type in women in 157 countries, and, in half of all BC cases, there has been found no association with specific risk factors [33]. These risk factors for BC include late age of childbirth or menopause after 50 years, with a prolonged estrogen exposure or the lack of breast tissue differentiation being possible causes [32].

BC has a wide genetic and clinical heterogeneity, with different subtypes and classifications. One of the most accepted BC classifications is based on immunohistochemical characteristics and hormone receptor expression (i.e., progesterone (PR), estrogen (ER) and human epidermal growth factor (HER2). Hence, the main four subtypes of BC are luminal A (ER+, PR+, and HER2−), luminal B (ER+, PR− or PR+, and HER2−), HER2 positive (ER− or ER+, PR− or PR+, and HER2+), and triple negative (ER−, PR−, and HER2−) [34].

BC treatment includes surgery, chemotherapy, radiotherapy and endocrine therapy, among others. Adjuvant therapy (e.g., radiation) after surgery is usually used to reduce the risk of local recurrence or metastasis [35]. There are many concepts involved in BC treatment decisions that are difficult to understand, including health risks and probabilities, technical medical information that is unfamiliar to most patients, and a multitude of options that can become overwhelming when accompanied by emotional factors, such as fear, when making treatment decisions [36]. Among other factors, BC subtype, stage of cancer, and patient health factor into treatment decisions. In addition, decisions regarding prevention, screening, and treatment have repercussions on future quality of life, which are difficult to predict [36].

#### 1.2.2. Breast Microbiome and Breast Cancer

The human breast microbiome is unique, and its composition differs from other parts of the body, having more α diversity (diversity within a single sample) and β diversity (diversity between different samples or ecosystems) than the skin microbiome (both microbiomes showing similar species relative abundances). Furthermore, clear differences exist between the breast microbiome in healthy and BC tissues, influencing the tissue microenvironment [37]. Some of the most abundant populations of bacteria observed in healthy breast tissue are *Enterobacteriaceae*, *Propionibacterium*, *Staphylococcus*, *Bacillus*, *Acinetobacter*, *Pseudomonas*, *Comamonadaceae*, *Gammaproteobacteria*, and *Prevotella*; existing essential differences between women from different countries [38].

Despite the high heterogeneity in breast microbiome, common changes in its composition are notable and may have deleterious effects. In this context, dysbiosis of breast and gut microbiome appears to influence BC metastasis through several mechanisms. In fact, some bacteria in breast tissue have been suggested to promote metastasis (e.g., *Staphylococcus aureus*, *Bacteroides fragilis*, *Fusobacterium nucleatum*, *Proteobacteria* spp.) [39]. Furthermore, the comparison of tissue samples (using 16S rDNA hypervariable tag sequencing, an alternative method to 16S rDNA amplicons for investigating the diversity and structure of prokaryotic communities) from patients with invasive BC and patients with benign breast disease has shown differences in microbial communities. A lower abundance of *Fusobacterium*, *Lactobacillus*, *Gluconacetobacter*, *Atopobium*, and *Hydrogenophaga* has been found to be associated with malignant disease [40]. Thus, it has been hypothesized that some of these bacteria could promote cancer development. For instance, *Enterobacteriaceae* and *Staphylococcus* may secrete genotoxins inducing DNA damage, or *Lactobacillus* could promote changes in TME, lowering pH through the production of lactic acid, which may increase the resistance of tumors to chemotherapy and radiation [37].

Breast tissues from donors with benign breast disease present an immunogenic microenvironment—higher infiltration of both innate and adaptive immune cells–in comparison with normal donors. Furthermore, later BC shows diminished B cell infiltration, suggesting that this phenomenon could have a preventive role in cancer progression [41]. These tissue microenvironment variations in stromal and immune cell composition have been associated with future cancer risk, and they could be explained, at least partially, by microbiome alterations [42]. For instance, the relative abundances of some bacteria, such as *Methylobacterium radiotolerans* in tumor tissue and *Sphingomonas yanoikuyae* in paired normal tissue, have been related to BC [43].

Finally, according to emerging preclinical data, dysbiosis of the breast microbiome may contribute to BC initiation and progression. Additionally, the breast microbiome may serve as a significant biomarker for treatment selection and prognosis [44]. The composition of the breast microbiome may differ depending on the subtype and severity of the disease. This may lead to immunosuppression, which allows tumor cells to evade the immune system [44].

#### 1.2.3. Gut Microbiome and Breast Cancer

The gut microbiome has been reported to be one of the environmental factors affecting BC development. One of the main mechanisms explaining this link would be the production of different metabolites by the gut microbiome, which may have effects in distant organs and tissues [45]. Key metabolites include short chain fatty acids (SCFAs), tryptophan, bacteriocins and phenylpropanoid-derived metabolites, all of which have been shown to impact cell division processes and immune signaling [17].

The development of BC is highly influenced by disturbances in estrogen metabolism [46]. This metabolism of estrogen molecules as well as the ECM transition processes appear to be influenced by specific gut metabolites [45]. For example, patients with breast tumors with HER2+ have a lower diversity of gut microbiome, with a higher abundance of *Bacteroidota* and a lower abundance of *Bacillota*, compared with those with breast tumors with HER2– [46]. Additionally, higher abundance of *Campylobacter*, *Streptococcus*, and *Moraxellaceae* has been observed in patients with BC (with and without bone metastasis), while patients with BC and bone metastasis showed the absence of *Megamonas* and *Akkermansia* [47].

**Figure 1:**
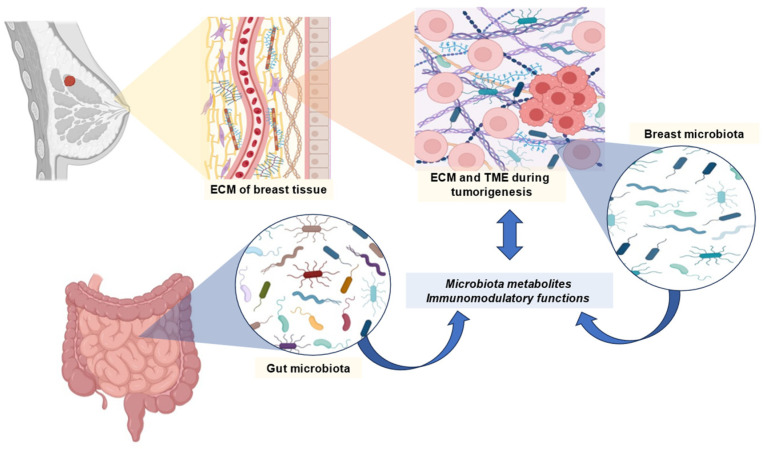
Overview of the relationship between microbiome and breast cancer. Abbreviations: ECM, extracellular matrix; TME, tumor microenvironment.

It is possible that BC is associated with an imbalance in the gut microbiome. As a result of these imbalances, there is a variation according to molecular type, stage and grade of cancer, menopause, menarche, body mass index, and physical activity. According to some studies, the gut microbiome may influence the effectiveness of BC therapies. BC is characterized by a loss of microbial diversity [48]. Among the species that are found, there is an increase in those that have deleterious effects, such as *Clostridium* and *Bacteroides*, and a decrease in those that are beneficial to health, such as *Faecalibacterium*, *Bifidobacterium* and *Akkermansia*. Furthermore, BC may be associated with a decrease in SCFA-producing bacteria and the levels of these metabolites [48]. A general overview of the previously mentioned relationships between BC and the microbiome is represented in Figure 1.

## 2. A Dual Role for Radiotherapy-Derived Metabolites in Disease and Treatment

### 2.1. Metabolites and Their Types

Radiotherapy affects the generation of metabolites by tumor-infiltrating cells and the microbiome both locally, in the tumor, and systemically. Several studies have demonstrated that ionizing radiation impacts the diversity of species that compose the microbiomes of several organs, such as the intestines [49], skin [50] and lungs [51,52,53]. The remodeling of the microbiome composition impacts the metabolites generated by these microorganisms, greatly affecting immune responses that influence the outcome associated with radiotherapy. As anticipated, SCFAs, including acetic acid, butyric acid, and propionic acid, exert effects in distant organs and are essential sources of energy for gut microbes. They play a number of regulatory roles in physiology and immunity in the host [54]. The molecule trimethylamine N-oxide (TMAO), which is produced by gut microbes, is also associated with host immunity [55]. Radiotherapy also affects the metabolism of tumor cells, immune infiltrating cells and adjacent cells, such as fibroblasts, impacting the TME and antitumor immune responses. In general, there are two ways by which ionizing radiation induces changes in the metabolites that compose the TME: (i) inducing metabolic changes in the cells from the TME to permit adaptation and survival and (ii) through the secretion of metabolites by dying cells.

### 2.2. Metabolites from the Microbiome and Immune Responses: Therapeutic Effects

**Figure 2:**
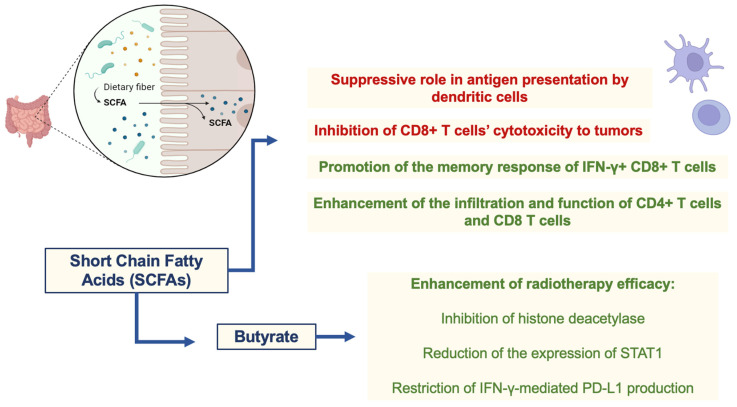
Dichotomic role of SCFAs in radiotherapy efficacy and immune cell modulation. Abbreviations: STAT1, signal transducer and activator of transcription 1; IFN-γ, gamma-interferon; PD-L1, programmed cell death ligand 1.

As cited previously, changes in the microbiome are known to affect immune responses locally and systemically. A critical player in these alterations is the metabolites liberated by microorganisms [56]. These metabolites are essential components of our diets, impacting immune cell function. SCFAs are well-known modulators of immune responses, with dichotomic effects that maintain homeostasis [57]. In this sense, SCFAs are the major metabolites influencing the efficacy of radiotherapy and related immune responses against tumors that can be enhanced after immunogenic cell death and neoantigen availability induced by ionizing radiation. Similar to their effects on immune responses, SCFAs, like propionate and butyrate, have a dichotomic role in radiotherapy efficacy. At least part of the effects of butyrate on enhancing radiotherapy efficacy can be attributed to its role in the cell cycle arrest of tumor cells through the inhibition of histone deacetylase and subsequent induction of FOXO3A, which regulates p21, p57, growth arrest and DNA damage-inducible 45 (GADD45) [58,59]. In addition, butyrate can reduce the expression of signal transducer and activator of transcription 1 (STAT1) through acetylation and can restrict IFN-γ-mediated programmed cell death ligand 1 (PD-L1) production in tumor cells (colorectal cancer). PD-L1 binds to PD-1 from T cells, leading to PD-1-dependent phosphatase activation and silencing of the T cell receptor (TCR)-downstream pathway and subsequent T cell activation. Therefore, inhibition of PD-L1 expression strengthens immune responses and restrains an important escape mechanism within tumors [60]. The duality of SCFAs on immune cells also seem to positively and negatively affect antitumor immunity associated with radiotherapy. In this sense, the suppressive role of butyrate in antigen presentation by dendritic cells, summed up by its supporting role on regulatory T cells (Tregs) function and metabolism, can impair antitumor immunity [61,62,63]. Furthermore, butyrate can restrict the effects of radiotherapy in a mouse model of colon adenocarcinoma (MC38-OVA) by inhibiting CD8+ T cell cytotoxicity to tumors [61]. On the other hand, butyrate can promote the memory response of IFN-γ+ CD8+ T cells [63], and SCFA-producing bacteria also positively affect the infiltration and function of CD4+ T cells and CD8+ T cells in BC patients [64]. Thus, the specific TME of each tumor seems to be a determinant of the role of metabolites in immune responses, followed by radiotherapy in a context-dependent manner. Nevertheless, the association of both probiotic (SCFA-producing microorganisms) and prebiotic (promotes the metabolism of SCFA-producing microorganisms) therapies with radiotherapy is an interesting therapeutic option to be evaluated in distinct tumors, as shown by different studies [51,52]. These studies suggest that various microbiome-based therapies, including probiotics, prebiotics, antibiotics, microbial metabolites, and natural compounds, may be effective in managing radiation-induced harm, as discussed later. The therapies described above provide a roadmap for managing radiation damage in the future, however, current research indicates that radiation adversely affects the gut microbiome, reducing the number of probiotic bacteria and their metabolite expression, which may contribute to the exacerbation of radiation-induced stomach damage [51,52]. SCFAs play a dichotomous role in radiotherapy efficacy and immune cell modulation, as shown in Figure 2.

#### Metabolites from the Tumor Microenvironment (TME) and Radiotherapy

As anticipated, radiotherapy affects the metabolism of cells from the TME. Cancer cells typically undergo death via apoptosis, ferroptosis, necroptosis, or necrosis, leading to the extravasation of specific metabolites, depending on the specific type of cell death. The surviving cells must adapt their metabolism to resist the negative consequences of ionizing radiation. These adaptations include anti-oxidative responses, cholesterol and nucleotide biosynthesis and DNA repair mechanisms [65]. Therefore, glutamine metabolism, which generates glutathione, a critical regulator of ROS levels, is enhanced in tumor cells that survive radiation treatment [65]. The pentose–phosphate pathway, which deviates from glycolysis, is also critical for nucleotide biosynthesis and DNA repair. Interestingly, both glutamine consumption and the presence of damaged DNA, followed by the subsequent release of type I interferons (IFN-I), will affect the immune responses in the TME. Not surprisingly, as with many aspects of the immune system, the outcome of metabolic changes in the TME and their related metabolites will vary depending on the context. Furthermore, ionizing radiation can cause significant endothelial cell damage, being associated with decreased nutrient supply to tumors and low oxygen tension (hypoxia) [65,66]. These changes also force the adaptation of surviving cells to the new TME after radiotherapy. Hypoxia will be associated with HIF-1α stabilization, which governs the transcription of several genes that are critical for metabolic adaptation. For instance, glutamine and glucose transporters are upregulated after hypoxia in an HIF-1α-dependent manner [65,66]. In this context, cancer cells exposed to hypoxia become metabolically reprogrammed, leading to a cellular response that is adaptive to hypoxia and resulting in tumor progression and metastasis [67].

### 2.3. Metabolic Pathways and Their Products: Therapeutic Effects

#### 2.3.1. Glutaminolysis and Lactate

Glutamine consumption is increased in tumor cells submitted to ionizing radiation, which is critical for survival. Glutamine-derived glutathione is essential for antioxidant responses and NADPH levels, and its reducing activity is an important resistance mechanism to ionizing radiation-associated ROS. Glutamine is also critical for nucleotide biosynthesis and DNA repair. Cancer-associated fibroblasts can release glutamine, supporting its consumption by tumor cells, subsequent lactate generation, TME acidification, and tumor cell survival [68]. As glutamine metabolism produces lactate, the consequences of lactate on immune responses become increasingly significant. Several studies have demonstrated the immunosuppressive effects of lactate, including (i) the metabolic support of both cancer-associated fibroblasts and Tregs, which restrict effector T cells’ antitumor activity [69,70]; (ii) promotion of the expression of programmed cell death ligand 1 (PD-L1) in tumor cells, inhibiting the activation of effector T cells after PD-1 binding [71]; (iii) impairment of type I interferon secretion and antigen presentation by dendritic cells, leading to Treg differentiation [72]; (iv) induction of macrophage polarization into a suppressive phenotype, supporting tumor cell metastasis [73]; and (v) blunting immune surveillance mediated by T cells and NK cells [74]. Lactate itself can also be consumed by tumor cells, being critical for their resistance to radiotherapy [75]. Interestingly, as with many other metabolites that modulate immune responses, lactate can also promote antitumor immunity in distinct circumstances. For instance, lactate can promote antitumor immunity mediated by memory CD8+ T cells expressing T cell factor 1 (TCF-1) [76]. Compellingly, supplementation with L-arginine has been found to enhance the antitumoral effects of radiotherapy in brain metastasis patients. L-arginine supplementation has been found to be associated with reduced lactate levels in tumors, mainly through the inhibition of glycolysis. This feature involves nitric oxide synthase 2-dependent GAPDH nitrosylation and PARP hyperactivation [77]. Therefore, the targeting of lactate receptors through agonistic or antagonistic receptors can probably synergize with radiotherapy and immunotherapy, depending on the cancer type. The major receptors of lactate are the G-protein coupled receptors (GPCRs) GPR81 and GPR132.

As cited above, glutamine is also a key component of the maintenance of cancerous cells’ survival and proliferation, by providing energy to various metabolic pathways, including the Krebs cycle and redox homeostasis, among others [78]. Under glucose deprivation, tumors produce NADPH from lactate and glutamine. IDH1 maintains redox balance in tumors by generating NADPH from lactate. By regulating the redox balance of tumors, glutamine supports the generation of NADPH and maintains the redox balance of NADPH [79].

#### 2.3.2. Mevalonate Pathway and Cholesterol: Use of Statins

Another metabolite that seems to be increased in the TME is cholesterol, synthesized de novo by the mevalonate pathway. Interestingly, inhibiting cholesterol synthesis with statins can be critical for a better prognosis in patients treated with radiotherapy [80]. The association of statins and radiotherapy can induce pyroptosis and apoptosis of tumor cells [81,82,83], and both types of cell death will be discussed later. This combination seems beneficial against lung tumors and glioblastomas [84,85] despite being uninvolved in the death of some glioblastoma cell lines submitted to ionizing radiation in vitro. These different outcomes associated with cholesterol biosynthesis modulation might result from its involvement in antitumor immune responses. This depends on the infiltrating T cells for each type of cancer. Regarding the effects of statins in BC, the evidence regarding this phenomenon is still scarce. However, statins have been associated with a reduced risk of major adverse cardiovascular events in patients with BC receiving radiotherapy, suggesting a protective effect of statins [86]. In accordance with this, there are studies involving the administration of statins to prevent anthracycline (NCT01988571) and anti-HER2 (NCT05559164)-related cardiovascular toxicity in BC patients. Additionally, some recruiting trials have proposed its application for BC therapy. For example, the multicentered double-blinded MASTER study (NCT04601116) aims to investigate the clinical outcome of adding statin treatment to the neoadjuvant therapy of ER+ BC patients. Another trial (NCT00816244) investigated the effects of statins as neoadjuvant therapy in postmenopausal BC patients, the results of which have yet to be reported.

Interestingly, cholesterol is known to modulate immune responses in many aspects. Membrane lipid rafts, enriched in cholesterol, are critical for cell signaling, organizing the T cell receptor (TCR) module along with CD3 chains and coreceptors (CD4 or CD8) [87]. Therefore, inhibiting cholesterol biosynthesis in T cells impairs proper TCR signaling and activation of T cells, probably restricting antitumor immunity [88]. In addition, cholesterol in the TME can increase angiogenesis and the checkpoint receptor expression in T cells, such as PD-1, by infiltrating T cells [89,90]. Oxygenated cholesterol (oxysterols), formed by the reaction of ROS with cholesterol, can also negatively affect T cell activation after interacting with the receptor LXR [91]. This is particularly important after radiotherapy since ROS and cholesterol are abundant after treatment [92,93]. On the other side, LXR–oxysterol signaling can inhibit myeloid-derived suppressor cell function, possibly promoting antitumor immunity in this case [94]. This duality of cholesterol in the TME and antitumor immune responses is also present in relation to tumor-associated macrophages and other myeloid cells, in which cholesterol crystals can lead to membrane stress-mediated NLRP3 activation and subsequent IL-1β and IL-18 release, which in turn promotes inflammatory responses and tissue damage, but also angiogenesis [95,96,97].

A major contributing factor to the development of BC appears to be the dysregulation of cholesterol homeostasis. Population-based epidemiological data and mechanistic studies in vivo and in vitro are needed to provide a more thorough understanding of cholesterol’s role in BC development and to develop better treatment and prevention strategies [98].

#### 2.3.3. DNA Damage and Type I Interferons

As discussed above, tumor cells must adapt their metabolism to promote DNA repair and survival. At the same time, extravasation of damaged nucleic acids (from mitochondria or the nucleus) into the cytosol can lead to the activation of different cytosolic pattern recognition receptors (PRRs), such as cyclic GMP–AMP synthase (cGAS), and subsequent type I interferon release. It has been shown elsewhere [99] that the role of type I interferons (IFN-I) in tumor biology is highly dichotomous. IFN-I can promote antitumor immunity through different mechanisms in certain situations, including (i) promotion of antigen presentation, optimizing T cell activation against tumor neoantigens [100]; (ii) induction of CD8+ T cell (cytotoxic T cell) survival and function, also enhancing their TCR sensitivity to its cognate MHCI peptide [101]; (iii) optimization of Th1 cell differentiation and antitumor immunity [102]; (iv) promotion of NK cell-mediated immune surveillance [103,104]; (v) restriction of the differentiation and recruitment of tumor-associated macrophages, indirectly preventing metastasis [105,106]; (vi) restraint of regulatory T cell (Treg—involved in the silencing of immune responses) recruitment, through the inhibition of chemokine (C-C) ligand 17 (CCL17) expression; and (vii) downregulation of vascular endothelial growth factor (VEGF) expression, restraining angiogenesis [106,107]. On the other hand, under different circumstances, IFN-I has also been described as promoting tumorigenesis by (i) supporting the glycolytic metabolism and function of Tregs [108]; (ii) inducing the expression of checkpoint receptors in effector T cells, silencing T cell-mediated immune responses and antigen presentation by dendritic cells (DCs) [102]; (iii) and promoting effector T cell exhaustion [109]. Therefore, more studies are necessary to understand the distinct factors present in each TME that influence the antitumor or pro-tumoral effects associated with ionizing radiation-mediated IFN-I release. The use of IFN-I for the treatment of viral diseases, like hepatitis B, provides evidence of the clinical benefits of recombinant IFN-I, and combining IFN-I with checkpoint receptor antagonists [110] or radiotherapy [111] can be an interesting perspective for the treatment of different tumors. Nevertheless, there are some issues that limit the clinical application of recombinant IFN-I and other strategies that elicit IFN-I responses [112]. Recombinant IFN or interferogenic molecules may exacerbate autoimmune or autoreactive conditions when delivered systemically. The use of intratumoral administration strategies is limited to injectable neoplasms, but excessive responses may also be elicited in cell types actively involved in anticancer immunity, such as cytotoxic T lymphocytes (which are highly sensitive to interferogenic stimuli). Furthermore, restoring potent IFN-I responses in the TME may not ultimately be sufficient to induce clinically meaningful anticancer immunity, requiring the development of multimodal therapeutic strategies that may include immune checkpoint inhibitors [112].

### 2.4. Metabolic Processes

#### 2.4.1. Metabolites from Common Genetic Defects in Tumor Cells

Some mutations in enzymes involved in distinct biosynthetic pathways, such as the citric acid cycle, may result in the accumulation of oncometabolites, which are specific metabolites produced by tumor cells in response to radiotherapy. The major oncometabolites are succinate, fumarate and 2-hydroxyglutarate (2-HG) [113]. Succinate can be recognized by its receptor succinate receptor 1 (SUCCNR1), which promotes several effects associated with metastasis, such as (i) angiogenesis, (ii) epithelial to mesenchymal transition, and (iii) tumor cell migration [113]. Furthermore, the presence of succinate might lead to enhanced glycolysis due to HIF-1α activation and subsequent increase in glucose transporters [114]. As with other metabolites, succinate also possesses a dichotomic effect in relation to antitumor immune responses, depending on the context. It can promote the release of IL-1β [115], a dual proinflammatory cytokine in tumor biology, while also restricting the secretion of IFN-I through the inhibition of MAVS signaling [116]. Furthermore, activation of SUCCNR1 in CD8+ T cells promotes T cell metabolic fitness and antitumor immune responses in mouse models of tumors [117]. Differently, succinate can restrict antitumor immune responses through metabolic impairment of T cells [118], while also promoting tumorigenesis through the induction of HIF-1α and the subsequent enhancement of glycolytic metabolism and angiogenesis in mouse models of BC [119]. Conversely, succinate-derived fumarate mediates succination, a post-translational protein modification, of gasdermin D, leading to resistance to cell death, i.e., pyroptosis [120]. As anticipated, succinate can be converted to fumarate, another oncometabolite, by the enzyme succinate dehydrogenase. Increased levels of intracellular fumarate lead to metabolic reprogramming, e.g., reversal of the urea cycle and argininosuccinate accumulation, making tumor cells dependent on arginine to sustain their metabolism and urea cycle [121]. Similar to succinate, fumarate also regulates immune responses, promoting IFN-I release [122] and antitumor immunity by cytotoxic CD8+ T cells [123]. In the opposite way, ZAP-70 succination restrains T cell signaling and subsequent activation, leading to dysfunctional effector T cells and inefficient antitumor immune responses [124]. Finally, 2-HG accumulation can promote tumor stemness, which is associated with resistance to different types of therapies [125]. Furthermore, 2-HG seems to be involved in inhibiting immune cell development from hematopoietic progenitors [113], also restraining T cell-mediated antitumor immune responses [126]. Therefore, the accumulation of 2-HG in the TME seems to contribute to the escape of tumor cells from antitumor immunity. The therapeutic modulation of these metabolites can be dependent on the cancer type. Agonistic or antagonistic antibodies against succinate receptor 1 (SUCNR1) or metabolic modulators that restrain the synthesis of the above-described metabolites are compelling treatment strategies to be combined with radiotherapy. Interestingly, metformin, an inhibitor of AMPK, has been observed to reduce intracellular levels of succinate [127] and is an interesting therapeutic option for adjunctive therapies against several tumors. For example, a combination of statins and metformin reduces the aggressiveness of glioblastoma [128], and their use in different types of cancer, especially in those that possess high levels of succinate and cholesterol in their TME, is a promising perspective to be evaluated. According to this section, succinate in the TME plays both a dichotomous and pleiotropic role, depending on the situation, while the inhibition of 2-HG seems to be a more reliable strategy by which to improve radiotherapy efficacy through antitumor responses.

#### 2.4.2. Metabolites from Dying Cells: Targets for Therapies

Tumor cells that do not adapt to the new microenvironment originating from ionizing radiation or that are sensitive to the radiation itself will die. In this sense, ionizing radiation has been described as being induced by different types of cell death in tumors and adjacent cells [129]. From 1972 to the late 1990’s [130] only two types of cell death were described: (i) necrosis, which occurs in an accidental, non-regulated manner, and (ii) apoptosis, which occurs in a regulated or programmed fashion. Necrosis is characterized by organelle swelling and loss of membrane integrity that is induced by different stimuli, like mechanical lysis of cells and diffuse membrane damage. Apoptosis happens after well-defined events that can be divided into intrinsic or extrinsic pathways. Both pathways induce a common pattern of morphological changes, which include cytoplasmic and chromatin condensation, followed by DNA fragmentation, cytoskeleton protein degradation and formation of membrane blebs, without loss of membrane integrity, known as apoptotic bodies [131]. The intrinsic apoptosis pathway is induced by a cascade of events initiated by increased mitochondrial membrane permeability, which is associated with cytochrome c release. Cytoplasmic cytochrome c binds to apoptotic protease activating factor 1 (Apaf-1), leading to the formation of an apoptosome composed of cytochrome c, Apaf-1, and procaspase 9 [132]. In the apoptosome, procaspase 9 is oligomerized and activated after autocatalysis and subsequently cleaves procaspase 3 into its active form, caspase 3. Caspase 3 is an effector caspase that cleaves multiple substrates, including beta-catenin; protein kinase C (PKC)-gamma and delta; poly(ADP-ribose) polymerase family member 16 (PARP-16); procaspase 9, in a positive feedback loop; and procaspase 7, leading to the activation of another effector caspase, caspase 7 [133]. Therefore, active caspase 3 is fundamental for the characteristic morphological changes and cell dismantlement in apoptosis [134]. As part of the apoptotic process, caspase 3 is an essential executioner molecule. Numerous studies have demonstrated a close association between caspase 3 expression and BC. However, caspase 3 expression remains uncertain in the context of BC prognosis [135].

The extrinsic apoptosis pathway is governed by cell signaling from membrane cell receptors, such as TNFR and Fas [136]. Engagement of these receptors with their respective agonists can lead to either (i) activation of proteins that possess death domains (DDs), such as TRADD, which subsequently lead to the recruitment and activation of other proteins that contain death effector domains (DEDs), like FADD, in the case of TNFR; or (ii), in the case of Fas, there is direct receptor-mediated activation of FADD and subsequently of procaspase 8 that interacts with FADD through the homotypic binding of DEDs [137]. The autocatalytic activity of procaspase 8 (initiator of apoptosis) leads to caspase 8 activation and, subsequently, the effector caspases 3/6/7 and apoptosis [138]. A decrease in AK2 function in BC leads to a reduction in the interaction of the AK2–DUSP26 complex with FADD, resulting in an increase in the levels of phosphorylated FADD in the nuclei of tumor cells, thus increasing tumor proliferation rates [139]. The presence of high levels of FADD expression or amplification in BC was found to be negatively correlated with the frequency of CD4+ T cells and the presence of dendritic cells. FADD mRNA expression was found to be significantly associated with recurrence-free survival in patients with BC. High levels of FADD expression have been frequently observed in luminal B and high-grade BC, which were found to be associated with shorter survival times free of metastases [140]. Clinicaltrials.gov lists five recruiting studies and four active and not recruiting studies investigating caspase 3 in the BC population. Meanwhile, there are no ongoing studies investigating FADD in BC patients.

#### 2.4.3. Other Types of Regulated Cell Deaths

Among other distinct types of cell death, we will discuss the concepts of those already described as being involved in ionizing radiation-mediated cell death beyond apoptosis and necrosis, i.e., (i) necroptosis, (ii) pyroptosis, and (iii) ferroptosis. Similar to necrosis, all of these share the loss of membrane integrity and induction of inflammatory responses [130], especially associated with danger-associated molecular patterns (DAMPs) release, such as HMGB1 and DNA. However, each regulated cell death has unique morphological and biochemical features. (i) Necroptosis is induced by different stimuli, mainly in the absence of caspase 8 activity and/or the cellular inhibitors of apoptosis (cIAP) E3 Ubiquitin ligase family of proteins [130]. In these conditions, autoactivation of the kinases RIP1 and RIP3 occurs, leading to the recruitment and activation of the pseudokinase mixed lineage kinase domain-like protein (MLKL) into the plasma membrane, where it mediates pore formation [141]. The complex RIP1/RIP3/MLKL is known as a necrosome. (ii) Pyroptosis is regulated by another complex of proteins in different conditions that leads to inflammasome assembly. The inflammasome complex of proteins, which distinct components can assemble, leads to procaspase 1/11 autoactivation and gasdermin D cleavage that drives plasma membrane pore formation and cell death [142]. Pyroptosis is also characterized by the formation of membrane blebs, known as pyroptotic bodies [142]. (iii) Ferroptosis is a form of cell death that depends on iron-mediated reactive oxygen species generation and lipid peroxidation driven by loss of activity of the lipid repair enzyme glutathione peroxidase 4 (GPX4) [143].

Researchers have recently studied not only the cytokines but also the metabolites associated with these distinct kinds of cell death. In this sense, the role of pyroptosis in wound repair, dependent on prostaglandin E2, can compensate for the proinflammatory and tissue-destructive role of IL-1β and IL-18 [144]. In this line, inhibition of cyclooxygenase 2 (COX2), involved in prostaglandin synthesis, can enhance both the immunogenic and tumor-destructive effects of pyroptosis, especially after radiotherapy. Similarly, apoptotic cells release several immunomodulatory molecules involved in silencing surrounding immune cells [145], restricting antitumor immune responses after radiotherapy. These metabolites, such as dihydroxyacetone phosphate (DHAP), fructose 1,6-biphosphate (FBP), guanosine monophosphate (GMP), inosine monophosphate (IMP), spermidine, adenosine monophosphate (AMP) and uridine 5’-diphosphate-glucose (UDP–glucose), when released together, but not individually, seem to regulate wound healing and anti-inflammatory responses [145]. Interestingly, not all of these metabolites are considered anti-inflammatory. After binding to its receptor P2Y14 (a G-protein coupled receptor-GPCR), UDP glucose leads to NLRP3 inflammasome formation and IL-1β and IL-18 release [146]. On the other hand, DHAP can, through prostaglandin J2 (PGJ2) [147], inhibit LPS-mediated TNF production and apoptosis of murine macrophages, while AMP, GMP and IMP are well-known agonists of several GPCRs involved in anti-inflammatory responses, and are possible targets for adjunctive immunotherapies associated with radiotherapy. FBP possesses anti-inflammatory effects, inducing adenosine secretion and adenosine receptor A2a activation (GPCR), which leads to the formation of cyclic AMP, CREB activation and the transcription of genes involved in anti-inflammatory responses, such as IL-10, and at the same time inhibition of proinflammatory genes induced by nuclear factor kappa B (NF-κB) [148,149]. All of these metabolites reinforce the anti-inflammatory role of apoptosis, being summed with the immune-silencing role of efferocytosis, mediated by the receptors of apoptotic bodies, which are also compelling molecular targets for therapies. These apoptotic body receptors can be diverse, with a common inhibitory function, such as Tyro Axl Mertk tyrosine kinase (TAM) receptors and T cell/transmembrane immunoglobulin and mucin domain containing 4 (TIM-4) [150]. The immunogenic death of tumor cells can enhance the effects of antitumor immune responses, as well as ameliorating the accessibility of tumor neoantigens for antigen presentation and subsequent T cell-mediated antitumor immunity. As a result, these types of cell death are typically linked to improved immunotherapy and radiotherapy.

## 3. Microbiome and Metabolites: A Systems Biology Approach

### 3.1. Omics Technologies: Genomics, Metabolomics, and Metagenomics

#### 3.1.1. Genomics and Metagenomics

There are approximately 150 times more genes in a microbiome than in the human genome. As a second genome, the microbiome is referred to as the metagenome [151,152,153]. In order to unravel the biological mechanisms of microbial modification of disease progression and responsiveness to therapies, including radiotherapy for cancer treatment, it is necessary to identify the factors that influence the composition of the microbiome. It has been established that the microbiome plays a significant role in the development of cardiovascular disease [154], cancer [155], respiratory disease [156], diabetes [157], inflammatory bowel disease [158], brain disorders [159], chronic kidney disease [160], and liver disease [161,162].

By generating high-throughput genomic data, omics technologies have changed the perspective in research. Metagenomics focuses on the genome of all of the microorganisms in a sample. Next-generation sequencing (NGS) approaches have enabled the analysis of all microbes without the need for cultivation since the development of the Human Microbiome Project (2009) [163]. The sequencing of 16S ribosomal RNA (rRNA) genes (16Sseq) and shotgun metagenome sequencing (MGS) are two of the most widely used methods. A 16Sseq experiment uses markers such as 16S rRNA, a conserved region, to distinguish between different species and estimate the composition of microbial taxa, while an MGS experiment entails the untargeted sequencing of the entire genome of all the microorganisms in the sample [164].

The advance in genomic sequencing technology through 16S rRNA and MGS has greatly facilitated research into microbial structures and diversity in their ecological niches, as well as their interactions within the commensal community and host–microbiome interactions. In recent decades, culture-independent methods based on NGS have significantly advanced our understanding of the microbiome, facilitating a deeper description of microorganism diversity [165].

The initial studies regarding the microbiome are relatively recent and have focused primarily on the digestive microbiome; however, given the variability of the microbiome across ecological niches in pathological contexts, it is of the utmost importance to identify specific patterns associated with clinical outcomes, such as responsiveness to therapies, to advance our understanding and improve patient care. An example may be found in Zeevi et al. [166]; as part of their study, the authors developed a machine learning algorithm that integrated blood parameters, dietary habits, anthropometrics, physical activity, and gut microbiome measured in the cohort and demonstrated that the algorithm accurately predicted postprandial glycemic response in real-life situations. A 100-person cohort was used to validate these predictions. As a result of blinded randomized control trials based on this algorithm, postprandial responses were significantly lower and gut microbiome structure was altered in a consistent manner [166].

As part of the Integrative Human Microbiome Project (iHMP), longitudinal studies have been conducted on omics datasets for both hosts and microbes [167]. During the ten-year project, reference sequences, multi-omics data sets, computational and statistical tools, and analytical and clinical protocols were developed as resources for the broader research community [168]. A baseline study of healthy adult subjects was used to characterize microbial communities from a variety of body sites (oral, nasal, vaginal, gut, and skin) and a series of demonstration projects were performed based on specific diseases or disorders [168]. During the second part of the study, three longitudinal cohort studies of representative microbiome-associated conditions expanded the repertoire of biological properties analyzed for both host and microbiome: pregnancy and preterm birth (vaginal microbiomes of pregnant women), inflammatory bowel disease (gut microbiome) and prediabetes (gut and nasal microbiome) [168].

It is imperative to adopt different computational analysis methods to integrate multi-omics data between the host and microbiome in order to impact personalized medicine. Obtaining host omics information and metagenome data, along with developing and incorporating more sophisticated analytical methods, will allow us to examine the mechanism of disease progression and facilitate the adoption of personalized medicine. By improving our understanding of this interaction, we will be able to unravel disease pathogenesis and solve the causality of host–microbe interactions [151]. Protocol standardization is one of the major challenges of microbiome research, particularly in terms of design, bioinformatics pipelines, and mixed biofilm studies. Some ecological models have been proposed to explain the complexity and interplay of microbial populations in order to address these issues. As well as producing mixed biofilms, these communities can be protected from the immune response of the host and from antimicrobial treatments. There is still a need for more studies using large sample sizes and standard and novel methods in order to understand the situation fully.

#### 3.1.2. Metabolomics

Metabolomics analysis is an important technique for detecting hundreds of small molecules simultaneously in a biological system. A number of low molecular weight compounds can be quantitated and qualitatively analyzed, including nucleic acid metabolites, carbohydrates, amino acids, vitamins, organic acids, and minerals produced by metabolism [151]. The technique uses gas chromatography–mass spectrometry (GC–MS), liquid chromatography–mass spectrometry (LC–MS), and nuclear magnetic resonance (NMR) spectroscopy to obtain label-free unbiased detection of all metabolites in a complex biological system [151].

It is possible to obtain information from metabolomics concerning the dynamic changes that occur during the development and progression of cancer. By identifying metabolites using cutting-edge metabolomics techniques, we will be able to identify biomarkers for early cancer detection, diagnosis, and treatment [169]. In population-based studies, metabolomics is increasingly being used to identify new etiological hypotheses and/or mechanisms related to BC development [170]. Despite its success in epidemiology studies, larger sample sizes, detailed information about menopausal status, BC subtypes, and repeated biological samples are required to facilitate comparisons between studies and to improve the validity of results, thereby allowing for clinical application [170]. As compared with non-obese BC serum samples, lipid, carbohydrate, and amino acid metabolism metabolites, oxidative phosphorylation, uric acid, and ammonia recycling vitamin metabolism (all of which contribute to ATP generation) are significantly higher in obese BC serum samples [171].

### 3.2. Multi-Omics Data Integration

DNA, RNA, proteins, and metabolites are key components of biological systems. These features influence signaling cascades and phenotypes when physiological or pathological conditions are present. High-throughput technologies have significantly enhanced the comprehensive study of molecular features at different levels, as previously discussed [165]. In order to integrate large datasets into a “Multi-omics” analysis from experimental and theoretical models that require advanced analytical tools, a system biology approach is required [172]. In spite of its computational complexity, network-based analysis is a valuable tool for analyzing and visualizing the relationship between variables, such as relationships between microbial compositions and gene expression [173].

As omics tools develop, they are becoming increasingly powerful methods of obtaining an impartial and integrated view of complex biological processes, such as the progression of a disease and the effectiveness of its treatment. As there have been no omics-level integration studies in this category, the methods have been limited to unsupervised clustering analyses based on correlation matrices between predefined groups [172,173]. To discover the role of the microbiome in human health, future microbiome analyses will have to integrate metagenomic data with data generated from the host genome, epigenome, transcriptome, and metabolome. For integrating data layers together, a set of best practices should be established to clarify the approaches that are most appropriate for each experimental design and allow for easier comparison of results across studies [174]. It is especially important to standardize microbiome analyses, which depend heavily on pipeline parameters and bioinformatics software [174]. Genetics research has benefited from the creation of large repositories, such as the Encyclopedia of DNA Elements [175] and the Cancer Genome Atlas [176]. These repositories aggregate genotypic and phenotypic data from multiple studies and can be used to perform large-scale computational analyses. In the context of microbiome multi-omics, developing and expanding such repositories would be beneficial, ensuring that pipeline parameters and batch effects are considered [177]. Furthermore, advances in large-scale sequencing, as well as artificial intelligence-related machine learning, can be used to analyze large scales of data related to microbes and determine the type and status of diseases [178]. In perspective, by integrating these cutting-edge technologies with genomics and metagenomics, more comprehensive studies of the microbiome’s role in cancer could identify novel therapeutic targets and develop precision medicine approaches to improve cancer outcomes.

## 4. Role of the Microbiome and Radiotherapy-Derived Metabolites in Breast Cancer

It was in 1970 that the first reports concerning bacterial metabolites and cancer with regard to aflatoxins were published [179]. Aflatoxins form an extremely potent group of carcinogens produced by *Aspergillus flavus*. Similar to *Fusarium* species, *Streptomyces hepaticus* produces potent carcinogenic mycotoxins [180,181,182]. The gut microbiome is involved in the off-target effects of radiotherapy, primarily due to the damage inflicted on the intestinal mucosa and subsequent toxicity. Radiotherapy reduces both the diversity and overall abundance of key bacterial populations such as *Bacteroidota*, *Enterobacteriaceae*, and *Bacillota*, while increasing the presence of *Fusobacterium* and *Proteobacteria* [183]. Interestingly, the role of the gut microbiome in the radiation response is dualistic. On the one hand, beneficial species such as *Lactobacillus sakei*, *L. acidophilus*, and *Bifidobacterium* spp. have been shown to possess radioprotective qualities [184]. On the other hand, radiation-resistant species such as *Deinococcus radiodurans* and *Rubrobacter radiotolerans* present resistance that can complicate treatment outcomes [185]. Studies suggest that *M. radiotolerans* and *A. radioresistens* are more common in estrogen-positive and postmenopausal BC patients [186]. Detecting inflammation-related markers and metabolites in cohorts may also allow actionable targets to be identified in order to enhance the effectiveness of antiangiogenic therapies currently used in various cancer types, including BC [187,188,189]. Additionally, radiotherapy simultaneously activates multiple pro-survival pathways, including those mediated by mutated ataxia telangiectasia, RAD3-related ataxia telangiectasia, AKT, extracellular-signal-regulated kinase, and nuclear factor kappa B, which promote DNA damage checkpoint activation and DNA repair, autophagy induction, and/or apoptosis inhibition [190].

TME significantly influences tumor response or resistance to radiotherapy. Metabolic changes induced by microbiome byproducts, which affect the balance between glucose utilization and fatty acid oxidation, play a crucial role in shaping the immune response within TME. These metabolic shifts have been shown to alter the radio sensitivity of BC cells [191,192]. Furthermore, metabolites derived from the gut microbiome, such as propionate, kynurenic acid (KYNA), and indole-3-carboxaldehyde, have demonstrated protective effects against the adverse side effects of radiotherapy, thereby improving the survival rates in BC patients [193]. The gut microbiome has also been found to regulate tumor responses to radiation therapy in BC differentially. In this context, targeting commensal fungi has been shown to enhance the efficacy of radiation therapy while reducing the expression of the C-type lectin receptor Dectin-1, a critical innate immune receptor involved in fungal sensing, which is associated with survival outcomes in BC [194].

Emerging therapeutic strategies, such as gut microbiome transplantation, have been seen to present a potential for mitigating radiation-induced toxicity. Fecal microbiome transplants (FMT) have improved survival rates and gastrointestinal integrity in radiated mice, offering a potential avenue for decreasing radiation-induced toxicity in BC patients [195]. Microbial metabolites, including the already discussed SCFAs, can affect the effectiveness of radiotherapy. It has been demonstrated that part of the antitumor effects of ionizing radiation are mediated by activation of immune cells [61]. The presence of butyrate in the gut as well as bacteria that produce butyrate inhibited radiation-induced antitumor response [61]. In tumor models treated with vancomycin, an antibiotic that targets butyrate-producing bacteria, the antitumor response was enhanced. In dendritic cells, butyrate inhibits the activation of the stimulator of the interferon genes responsible for activating the cytotoxic T cells that destroy tumors [61]. In the TME, butyrate derived from gut bacteria inhibited the induction of interferon by ionizing radiation [196]. Radiation-induced antitumor mechanisms were restored when butyrate-producing bacteria were removed [196]. Additionally, melatonin has been found to prevent radiotherapy side effects by activating microbiome-linked signaling pathways [197]. While there is growing evidence that the gut microbiome influences BC radiotherapy outcomes, further research is needed to elucidate its full potential, particularly studies with large sample sizes that integrate microbial, metabolic, and variable factors from BC patients. Together, SCFAs and butyrate have been discussed in terms of their promotion of anti-inflammatory responses and their enhancing of the efficacy of immunotherapies [198,199]. This interplay highlights the potential of targeting microbiome-related metabolites to improve cancer treatment outcomes. Thus, the development of microbiome-based therapeutic interventions could pave the way for more effective and personalized treatments for BC patients [200].

## 5. Dietary Therapeutics

### 5.1. Prebiotics and Probiotics: The Modulation of the Gut Microbiome

Prebiotics and probiotics play a pivotal role in cancer treatment, particularly when combined. Probiotics produce a variety of metabolites that inhibit inflammation and enhance anti-tumor immunity, while prebiotics serve as essential substrates that support the production of these metabolites by probiotics [23,201]. Prebiotics, often referred to as functional foods, are indigestible components of food that benefit the host by selectively stimulating the growth and/or activity of specific bacteria in the colon [202]. Dietary fibers, such as inulin, resistant starch, and lactulose, have been found to regulate the composition of the intestinal microbiome, potentially mitigating the gut microbiome imbalances that arise during radiotherapy [203].

Probiotics contribute to microbial translocation, enhance the function of the gut mucosal barrier, and exhibit antipathogenic and anti-inflammatory properties, all of which reduce tumor formation and metastasis. Probiotics demonstrate antimutagenic and anticancer properties by modulating the gut microbiome, binding and degrading carcinogenic compounds, and producing antioxidants and anti-inflammatory molecules, making them a valuable adjunct in BC radiotherapy [204].

Combining probiotics and prebiotics to enhance current cancer therapies is a novel approach in the field. A number of studies have demonstrated the importance of symbiotics for the control of intestinal microecology and for the prevention and treatment of cancer. According to these findings, symbiotics may become possible microecological modulators for adjuvant cancer treatment [205]. Indeed, symbiotics may decrease long-term cancer risk by fostering a robust and health-promoting gut microbiota that lowers the risk of cancer development and recurrence [206].

Certain probiotic strains, such as *Lachnospiraceae* and *Enterococcaceae*, promote hematopoiesis and protect against gastrointestinal damage. Additionally, studies by Riehl et al. have shown that *Lactobacillus rhamnosus GG* protects the small intestinal epithelium from radiation-induced injury [207]. Models of intestinal radioprotection have been developed using cell lines and enteroids, as well as by analyzing clinical outcomes and crypt survival in vivo. In order to evaluate tumor radioprotection, fractionated abdominal radiation and single-dose radiation were used together with syngeneic CT26 colon tumor grafts [207]. In addition to releasing radioprotective lipoteichoic acid, *Lactobacillus rhamnosus* GG acts as a “time-release capsule”. By activating macrophages and prostaglandin-E2 secreting mesenchymal stem cells, lipoteichoic acid primes the epithelial stem cell niche to protect epithelial stem cells [207]. However, gastric acid and ionizing radiation may compromise probiotics and fecal microbiome [208].

Furthermore, probiotic strains, including *Lactobacillus* and *Bifidobacterium*, have been proven to be both safe and effective in mitigating the adverse effects of chemotherapy and radiotherapy while also enhancing the immune response. Probiotics represent a promising therapeutic strategy for preventing and treating complications in immunocompromised cancer patients [209]. A multi-strain probiotic formulation was found to be safe and well tolerated in a chronically ill cohort undergoing oncological treatment. This formulation alleviated symptoms such as diarrhea and constipation while maintaining stool consistency and frequency during chemotherapy and radiotherapy. Importantly, intestinal dysbiosis, characterized by decreased microbial diversity and increased pro-inflammatory species, was not observed, suggesting that probiotic supplementation may help prevent dysbiosis during cancer treatments [210]. Moreover, probiotic consortia have been shown to attenuate radiation-induced intestinal injury by modulating the gut microbiome and metabolites, reducing inflammatory symptoms, and regulating oxidative stress. These findings underscore the potential of probiotic-based therapeutic strategies for maintaining intestinal health during radiotherapy [211]. Although there are relevant completed clinical trials about pre/probiotic-based therapies in BC, including those, for instance, that investigated the breast tissue microbiota and inflammatory markers of female patients receiving probiotics containing *Lactobacillus rhamnosus* GR-1 and *Lactobacillus reuteri* RC-14 (NCT03290651) or which studied how probiotics would affect CD8+ T cell infiltration in the TME of BC patients receiving a cocktail of *Saccharomyces boulardii*, *L. plantarum*, *L. rhamnosus*, *L. casei*, *L. salivarius*, *L. acidophilus*, *L. brevis*, *L. paracasei*, *B. subtilis*, *Bifidobacterium lactis*, *B. bifidum*, *B. breve* and *B. longum* (NCT03358511), none have reported their results. Another recruiting study (NCT06631092) proposes to investigate the intestinal microbiome of triple-negative BC patients who submitted the neoadjuvant therapy based on the neoantigen-targeting cancer vaccine NECVAX-NEO1, associated with immunotherapy (anti-PD-1 monoclonal antibody) and chemotherapy (epirubicin, cyclophosphamide and/or nab-paclitaxel). Interestingly, several recruiting clinical trials focus on improving the quality of life of BC patients and survivors. For example, there is a trial to study the gut microbiome and treat chemotherapy-induced alopecia in BC patients by administering the synbiotic formula BLHK03 (NCT06560385), which is the first study investigating interactions between probiotics and drugs in BC patients undergoing chemotherapy. Another recruiting trial focuses on examining the effects of probiotic bacteria from Kombucha, a fermented drink, on BC patients’ sleep quality and anxiety (NCT05717972). Furthermore, there is a study (NCT04784182), though with a significant limitation of only three participants enrolled, that proposes to investigate anxiety, inflammatory markers, and the fecal microbiome composition of BC survivors supplemented daily with probiotic *Lactobacillus helveticus* and *Bifidobacterium longum*, and prebiotic containing fructooligosaccharides (FOS). Hence, no studies have demonstrated the efficacy of pre/probiotic administration in treatment outcomes in BC patients, which is a critical opportunity to investigate the possibilities involving immune activation associated with BC immunotherapy. To integrate pre-, pro-, and synbiotics into cancer treatment in the future, large studies with appropriate doses and timing are essential to gain a comprehensive understanding of the complex interplay between the gut microbiome and cancer biology.

### 5.2. Interventions in the Diet: Dietary Factors

Although dietary modifications may alter the composition of the gut microbiome, the evidence suggests that diet interventions may reduce toxicity, improve chemotherapy efficacy, and reduce the risk of long-term complications in cancer patients, although the data are sparse [212]. Changes in the gut microbiome following dietary adjustments occur rapidly and are reversible, indicating that their effects may be transient. Consequently, research is increasingly focused on identifying ways to prolong and maintain these effects, as well as uncovering the underlying mechanisms involved in this interaction [213]. The results of epidemiological studies indicate that people who practice intermittent fasting for religious or personal reasons have a longer life expectancy and a lower incidence of cancer and cardiovascular disease. Researchers have tested the effects of fasting in several preclinical models of cancer initiation and progression, with mixed results [212,214,215]. Though calorie restriction regimens vary, convincing evidence has been found that calorie restriction can delay BC in both spontaneous and carcinogenesis models [215,216,217,218]. There has been great interest in using a ketogenic diet as an alternative to fasting and caloric restriction. Ketogenic diets have a longstanding safety record as a treatment for epilepsy and may be more tolerable in some patients. A recent meta-analysis compared the unrestricted ketogenic diet with a standard diet in murine cancer models and concluded that the ketogenic diet caused an overall tumor growth delay [219]. Some studies have examined the effects of a ketogenic diet during anticancer treatment, reporting synergy in most cases with irradiation, metformin, and chemotherapy. One of these was performed using BC cells, and the results are related to the reduction of tumor growth [220].

Given that oxidative stress and inflammatory responses are the primary pathogenic pathways for radiotoxicity, interventions such as caloric restriction and fasting may hold the potential to mitigate radiation-induced intestinal damage [221]. These dietary restrictions may help counter gut dysbiosis, improving host metabolism and immune response. Specifically, disruptions in gut permeability and bacterial translocation from the lumen to the mucosa can negatively influence immune function. Intestinal immune homeostasis is regulated by the interplay between epithelial and dendritic cells, and dietary modulation of the gut microbiome could enhance the effectiveness of radiotherapy in cancer patients. Nevertheless, these findings remain preliminary, and clinical trials are required to substantiate their clinical relevance [222]. While no definitive conclusions have been drawn regarding the efficacy of dietary interventions, an individualized approach is recommended. Tailored dietary strategies, informed by professional guidance, can help manage symptoms that emerge during radiotherapy treatment, providing a more targeted approach to care [223].

## 6. Social and Ethical Considerations

The integration of microbiome research into cancer treatment introduces a range of ethical considerations. These issues include obtaining proper patient consent, managing the potential unintended consequences of microbiome manipulation, and ensuring equitable access to microbiome-based therapies across all socioeconomic groups. With the increasing focus on personalized medicine, it is crucial to guarantee that such innovative treatments are not limited to specific populations but instead made available to all patients. Patient perspectives play a key role in the success of microbiome-based interventions. Patients may have varying levels of comfort regarding such treatments, especially with procedures like FMT. Patient education about the microbiome’s role in cancer therapy is vital for ensuring informed consent and encouraging adherence to treatment protocols. By engaging patients in meaningful discussions about these novel therapies, healthcare providers can address concerns, enhance patient understanding, and ultimately improve the overall treatment experience. These ethical dimensions not only require careful consideration by healthcare professionals but also emphasize the importance of comprehensive patient education and involvement in decision making to foster trust and enhance treatment outcomes [224].

## 7. Research Priorities and Future Directions

BC death rates have decreased with earlier detection and improved treatments, but BC remains the most common cancer among women, and incidences are expected to continue to rise [225]. According to current projections, the number of new BC cases is increasing rapidly and will reach 22 million by 2030, with the majority occurring in low- and middle-income countries [226]. To combat this problem, multiple strategies must be employed.

### 7.1. Studying Interdisciplinary Topics

The recent focus on early detection, prevention, and risk reduction may have contributed to the reduction in the number of BC-related deaths. Using data strategy and advanced analytics to minimize treatment toxicity has been one of several novel strategies adopted by several governments to improve cancer survivorship and transform cancer research [227]. As a result of technological advancements and scientific breakthroughs, progress against BC has accelerated. This progress has manifested in increased accessibility to affordable genome sequencing [228], targeted immunotherapies [229], mRNA vaccines [230], artificial intelligence [231], novel circulating biomarkers [232], in vivo CRISPR screening [233], among others. By addressing the intersections between socio-economic, geographic, and demographic factors, BC health equity has been improved globally [234]. Several studies have shown that their participants place a high priority on quality-of-life issues, with side effects of surgery, endocrine therapy, and chemotherapy being central topics of conversation [235]. In addition, there was found to be a significant emphasis placed on the psychological impact of a BC diagnosis and survivorship, as patients felt that these areas were under-researched and that improvements could be made [236]. To advance knowledge and improve patient outcomes, a holistic understanding and approach are required, one in which the aforementioned variables contribute to a comprehensive picture of the BC [237]. Artificial intelligence and machine learning are essential to understanding the relationship between all of the variables discussed in this topic. This is achieved through the construction of robust models, which can be generalized and are free from bias, and explainable machine learning for multifactorial longitudinal data to assess in a more accurate manner and to ensure future research success [238].

The objective of scientific research is to systematically compare practical experiences with existing literature to provide empirically grounded clinical guidance and meticulously adhere to the principles of evidence-based medicine in all clinical settings. The implementation of such a rigorous approach has the potential to significantly contribute to the establishment of uniformity across diverse socio-economic contexts [237].

### 7.2. Methodologies Based on Innovation

In BC research, some innovative methodologies may be applied to the study of the etiology of heterogeneity in tumor subtypes, such as risk factors among different ethnic groups and tumor subtypes. It is imperative that these methods are continued, particularly for patients with ER- or basal-like tumors for which few effective treatments are currently available [239]. There is a need to investigate the mechanisms underpinning known risk factors, including the reasons for heterogeneity by menopausal status or tumor subtype, by utilizing emerging technologies (e.g., metabolomics and proteomics) to assess local and systemic biomarkers as well as tumor heterogeneity [240]. To improve and validate risk prediction models, it is necessary, for example, to include both biomarkers (e.g., breast imaging, genetics, and hormones) and lifestyle factors [241].

Additionally, models need to be developed that can be more accurately applied at both the youngest and oldest ages, among diverse ethnic groups, and for subtypes, especially the ER subtype, for which models perform less well [242]. Additionally, it is crucial to identify how to successfully implement known (e.g., weight maintenance or reduction) and future preventive strategies during vulnerable times [243].

Physical exercise has been added to the treatment of BC as an essential and innovative complement to traditional methods. Observational epidemiological evidence has consistently shown that physical activity and weight control reduce the risk of BC. However, questions remain about the causality of this relationship and whether confounding factors could influence these associations [244].

Physical exercise is critical in enhancing physiological processes, such as cardiovascular fitness, muscular strength, and psychological wellbeing, which are often negatively impacted by BC treatment [245]. Chemotherapy and radiotherapy are associated with declines in physical performance, increased body fat, and reduced lean mass, as well as elevated risks of depression and anxiety. Studies have shown that structured exercise interventions, including both aerobic and resistance training, can effectively counter these effects. These programs have been demonstrated to improve physical fitness, maintain lean body mass, enhance self-esteem, and reduce fatigue without significant adverse events, thus emphasizing their safety and efficacy during cancer recovery [246,247].

Moreover, systematic reviews and meta-analyses have reinforced the idea that exercise is vital in improving quality of life and managing treatment-related side effects such as fatigue and psychological distress. Resistance training, in particular, is beneficial for maintaining bone health, which is crucial for patients at risk of osteoporosis due to BC treatments. These findings collectively highlight that integrating exercise as a core component of BC rehabilitation can mitigate the adverse effects of treatment on physical and psychological health, ultimately improving the prognosis and overall quality of life for survivors [245,248].

According to the above, exercise interventions are a valuable strategy for preventing and treating cancer by preventing the decline in physical fitness (e.g., cardiorespiratory fitness and strength). Resistance (66% 1 RM (maximum repetition) and aerobic (65% VO_2_ max) training and combined interventions provide the most evidence about the benefits of systematic physical activity in people with BC [249]. In addition, physical exercises seem to modulate the microbiome, and investigating the role of microbiome change in the benefits of exercise is an interesting aspect to be evaluated [250].

### 7.3. Funding and Policy

Research funding priorities must be reviewed considering factors such as incidence, prognosis, and public perception of the need for funding different types and stages of BC, as well as investment analyses [251,252]. If funding is redistributed, multidisciplinary stakeholders, including patients, will need to establish research priorities to ensure that research addresses critical issues of concern to the cancer community. In the area of cancer and health, limited resources are available for research and development investments. Investing wisely is essential [251,252].

## 8. Conclusions

The gut microbiome plays an important role in the development of BC. As a result of several mechanisms, intestinal dysbiosis may favor certain microorganisms in our intestines that contribute to the development of certain types of BC. Local and systemic immune responses can be affected by changes in the microbiome. Metabolites released by microorganisms that compose the microbiome play an essential role in these alterations. In addition to being important components of our diet, these metabolites also profoundly impact the function of our immune system. In this context, there is evidence that SCFAs have dichotomous effects on immune cells in the context of radiotherapy, affecting antitumor immunity both positively and negatively. To resist the adverse effects of ionizing radiation, the surviving cells must adapt their metabolism. These adaptations include antioxidative responses, cholesterol and nucleotide biosynthesis, and DNA repair mechanisms. Therefore, radiation-surviving tumor cells display enhanced glutamine metabolism, which produces glutathione, an essential regulator of ROS. The mevalonate pathway also synthesizes cholesterol de novo in the TME. The extravasation of damaged nucleic acids (from mitochondria or the nucleus) can trigger the activation of cytosolic PRRs, such as cGAS, and the release of type-I IFNs. Succinate, fumarate, and 2-HG are the principal oncometabolites. All these metabolic pathways and their related metabolites have been described as participating, in a dichotomic fashion, in both tumor biology and immune responses, and are worthy of evaluation as targets for therapies in BC.

In BC, omics tools have continued to become powerful tools for obtaining an objective and integrated view of complex biological processes. Future microbiome analyses must integrate metagenomic data with genotypic, epigenomic, transcriptomic, and metabolomic information to better understand how the microbiome contributes to human health. It is essential to integrate data layers together to clarify which approach is suitable for each experimental design and facilitate comparisons between studies. It is particularly important if we are interested in standardizing microbiome analyses, which are highly dependent upon pipeline parameters and bioinformatics software. Artificial intelligence and machine learning are essential for understanding the relationship between all of the variables discussed.

Emerging therapeutic strategies, such as gut microbiome transplantation, can mitigate radiation-induced toxicity. Dietary interventions with pre- and probiotics, changes in diet, and physical exercise are novel strategies in treating BC. Finally, it is imperative to develop a holistic understanding and approach to BC to advance knowledge and improve patient outcomes.

## Data Availability

Not applicable.
